# Reirradiation of Whole Brain for Recurrent Brain Metastases: A Case Report of Lung Cancer With 12-Year Survival

**DOI:** 10.3389/fonc.2021.780581

**Published:** 2021-11-26

**Authors:** Minmin Li, Yanbo Song, Longhao Li, Jian Qin, Hongbin Deng, Tao Zhang

**Affiliations:** Department of Oncology, The First Affiliated Hospital of Chongqing Medical University, Chongqing, China

**Keywords:** reirradiation, whole brain radiotherapy, lung cancer, brain metastasis, long survival

## Abstract

Whole brain radiotherapy (WBRT) for brain metastases (BMs) was considered to be dose limited. Reirradiation of WBRT for recurrent BM has always been challenged. Here, we report a patient with multiple BMs of non-small-cell lung cancer (NSCLC), who received two courses of WBRT at the interval of 5 years with the cumulative administration dose for whole brain as 70 Gy and a boost for the local site as 30 Gy. Furthermore, after experiencing relapse in the brain, he underwent extra gamma knife (GK) radiotherapy for local brain metastasis for the third time after 5 years. The overall survival was 12 years since he was initially diagnosed with NSCLC with multiple brain metastases. Meanwhile, each time of radiotherapy brought a good tumor response to brain metastasis. Outstandingly, during the whole survival, he had a good quality of life (QoL) with Karnofsky Performance Score (KPS) above 80. Even after the last GK was executed, he had just a mild neurocognitive defect. In conclusion, with the cautious evaluation of a patient, we suggest that reirradiation of WBRT could be a choice, and the cumulative radiation dose of the brain may be individually modified.

## Introduction

Brain metastases (BMs) occur in 40% of patients with systemic cancer, among which lung cancer is the most common primary tumor (1). Radiation therapy is the cornerstone of modern brain metastases treatment (2), and whole brain radiotherapy (WBRT) is the standard option for multiple BMs. As a result of advances in systemic therapy management, there shows greater rates of recurrence of BMs, as patients with lung cancer have been living longer.

The management of recurrent cranial metastatic disease previously treated with WBRT represents a challenge owing to the potential high risk of radionecrosis and neurocognitive deterioration, especially when choosing reirradiation of WBRT (3). The reported data on the topic of retreatment with WBRT is fractured and without a clear consensus (4, 5).

In this report, we presented a case of a patient with multiple BMs of NSCLC who underwent repetitive WBRT treatment during the course of the disease. The cumulative administration dose for the whole brain was 70 Gy, and a boost for the local site was 30 Gy. Meanwhile, during the whole survival, he had a good quality of life (QoL) and mild neurocognitive defects. Then, we discussed how we chose the strategy of reirradiation for brain metastasis.

## Case Presentation

A 42-year-old Chinese man presented with cough, sputum, and facial paralysis in May 2006. The fibrobronchoscope demonstrated adenocarcinoma at the site of the inferior lobe of the left lung. At the same time, the initial magnetic resonance imaging (MRI) showed that the tumor had already invaded the brain with multiple intracranial metastases. The clinical staging was stage IV (cT4bN2M1 IVb), so surgery was not indicated. After careful evaluation, we suggested a combination of chemotherapy and radiotherapy as the optimal therapy for him. After one cycle of Gemcitabine and Cisplatin (GP) chemotherapy has been completed, the patient underwent radiotherapy for the primary site with a total dose of 70 Gy in 35 fractions, using the two-dimensional radiotherapy (2DRT) technique. The WBRT with a dose of 40 Gy in 20 fractions was delivered initially using 2DRT with two opposing lateral fields and a rotated collimator to fit the whole brain to shield the lens. Subsequently, we measured the depth and size of metastatic sites on diagnostic CT and marked them on the body. Then, a boost plan that employed an anterior field and a lateral field with a 45° wedge was delivered for intracranial metastatic sites with 10 Gy in five fractions based on these marks and measurements. After that, he was treated with three cycles of TP (Paclitaxel and Cisplatin) chemotherapy. After the treatment, the symptoms alleviated apparently, and the serological tumor markers decreased apparently. The radiographic tumor response was evaluated as a complete remission (CR). In addition, he had no prolonged fatigue and/or neurocognitive defects. It is a pity that because it has been a long time, he could not supply his medical information in details.

**Figure 1:**
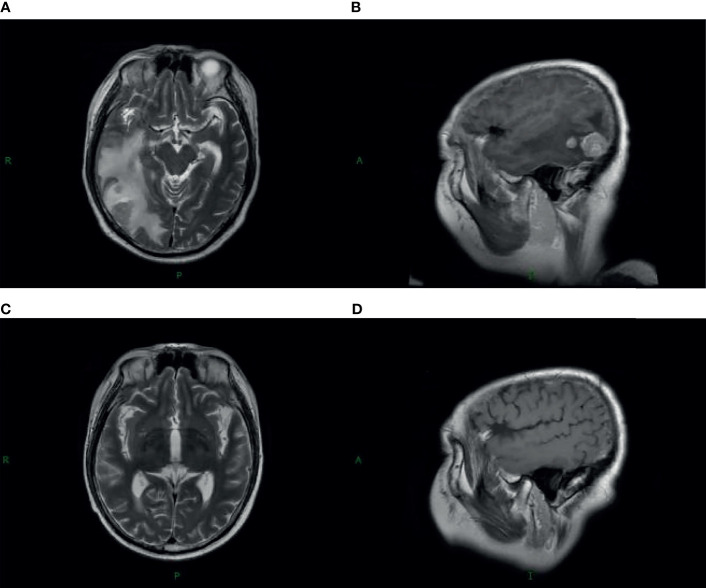
Magnetic resonance imaging (MRI) with contrast enhancement of brain metastasis. **(A, B)** July 2011, before radiotherapy on brain. **(C, D)** September 2011, after radiotherapy on the brain.

Without regular follow-up, the complete radiological remission lasted almost 5 years; in July 2011, the patient experienced the first intracranial relapse of multiple lesions, located in the right temporal lobe, parietal lobe, occipital lobe, and the left frontal lobe regions (**Figures 1A, B**), which had already received a boost of radiotherapy in 2006. He was found to have disturbance of consciousness by his family. The routine scan of the whole body showed that extracranial lesions were stable. Even though the patient’s epidermal growth factor receptor (EGFR) mutation status was unknown, he chose to accept erlotinib 150 mg daily. Meanwhile, he received reirradiation of the whole brain with a dose of 30 Gy in 15 fractions adopting three-dimensional radiotherapy (3DRT). The patient was immobilized with a thermoplastic mask. We contoured the target volume on the fusion images of CT/MRI and delivered a single isocenter coplanar intensity-modulated radiation therapy (IMRT) boost plan with a dose of 20 Gy in 10 fractions for metastatic intracranial tumors. Then, four cycles of nimustine (125 mg per 3 weeks) and maintenance erlotinib were followed. A follow-up MRI scan in September 2011 revealed that metastatic brain lesions regressed significantly (**Figures 1C, D**).

**Figure 2:**
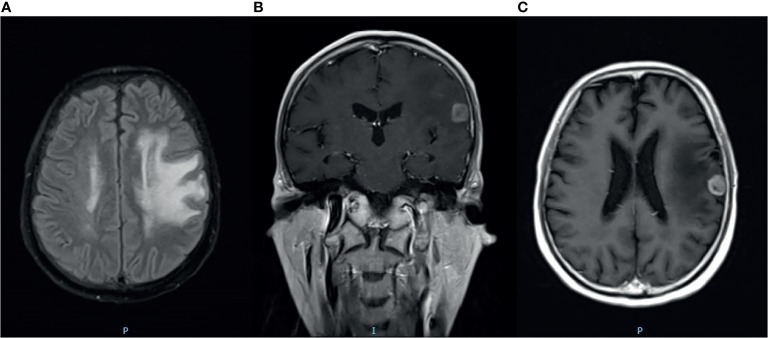
Magnetic resonance imaging (MRI) with contrast enhancement of brain metastasis. **(A–C)** January 2016, before GK radiotherapy on the brain.

**Figure 3:**
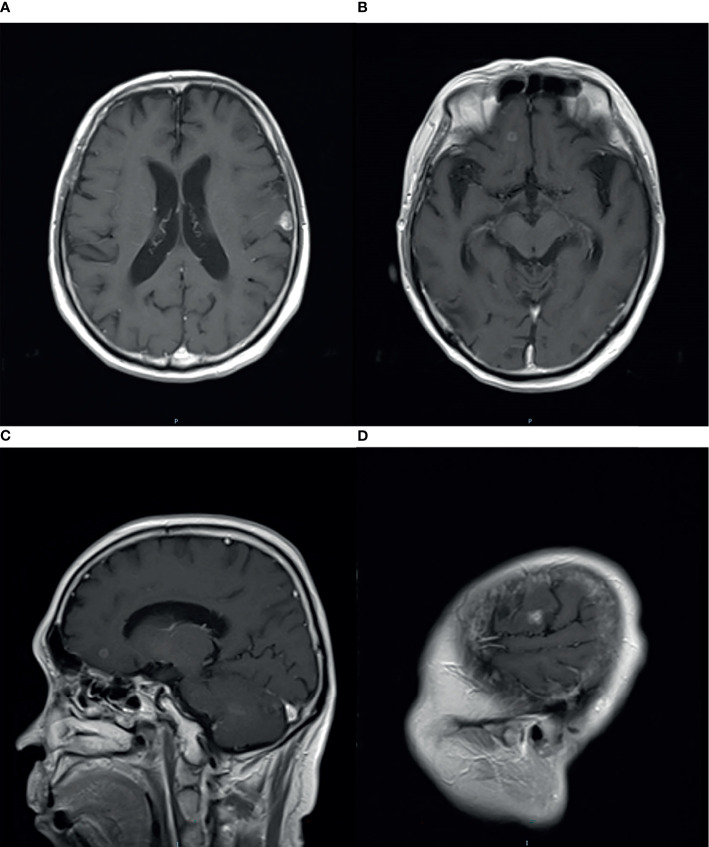
Magnetic resonance imaging (MRI) with contrast enhancement of brain metastasis. **(A–D)** April 2016, after GK radiotherapy on the brain, new intracranial lesions have been observed.

There were no signs of progression until January 2016, when a relapse was located on the left parieto-temporal lobe (**Figures 2A**–**C**). Then, GK radiotherapy was performed focusing on the intracranial single lesion of the patient, which was executed at the Third Military Medical University Hospital, China, with an 18-mm collimator. The central dose of the target was 25.4 Gy, and the isodose line of 14 Gy (55% of the central dose) covered the whole tumor. The tumor response of this patient was a partial response (PR) at initial treatment, but just after 3 months, other new intracranial lesions have been observed in the frontal lobe (**Figures 3A**–**D**). Due to tumor progression in such a short time after radiotherapy, we chose chemotherapy with nimustine (125 mg per 4 weeks) as the palliative treatment modality. From then, recurrent pulmonary infection bothered the patient, and he eventually died of respiratory failure in August 2018.

Outstandingly, during the whole survival, the patient had a good QoL with Karnofsky Performance Score (KPS) above 80. Even after the last GK was executed, he had just a mild neurocognitive defect in March 2018 after neuropsychological testing including 13 questionnaires in the Department of Geriatrics (**File 1**). Together with the brain MRI (slight necrosis and cerebromalacia), we found that the reirradiation of the whole brain did not bring severe neurocognitive defects as we worried before. It is noteworthy that no serious toxicity was observed throughout the entire survival, although the administered dose was far beyond the limits.

## Discussion

Brain metastasis management is a complicated task. In this case, we chose WBRT as the first-line therapy, as it is the conventional option for multiple BMs. Generally accepted treatment for WBRT is 30 Gy in 10 fractions (6, 7). Additional radiation doses may be executed depending on the patient prognosis. Because this patient was diagnosed initially at 43 years old, we finally chose WBRT with a dose of 40 Gy in 20 fractions and a boost for intracranial metastatic sites with 10 Gy in 5 fractions, considering his life expectancy.

Treatment should be decided individually, and the appropriate patient selection is very important to benefit from reirradiation. It is most essential to evaluate the prognosis of patients with BMs. The prognostic index that has now become the most prominent is the diagnosis-specific Graded Prognostic Assessment (DS-GPA) (8). As defined by DS-GPA classes, our patient is a male of younger age (<65 years) with a good KPS (KPS >70), control of primary disease, and absence of extracranial metastases. Theoretically, even though the EGFR mutation status was unknown, he was supposed to deserve a better prognosis. Thus, when he had a brain relapse, we chose a positive strategy for him.

For recurrent cranial metastatic disease, the optimal treatment option is not clear. There is an increasing number of reports on the topic of reirradiation with WBRT, but still no clear consensus has been reached. Radiotherapy oncologists agreed that several factors must be considered simultaneously in the process of selecting patients (9, 10), including previous treatment details, such as dose, fractionation, volume, and the interval between the two irradiations, and the current patients’ condition, such as the cranial lesions’ number, location, and size, patient’s performance score, the status of extracranial disease, and patients’ life expectancy. One of the earliest, large retrospective cohorts of patients undergoing repeat WBRT was conducted by Cooper et al. in 1990 (11). He concluded that reirradiation with WBRT for cerebral metastases is a viable option for patients who experience cranial relapse more than 4 months after a satisfactory response to initial WBRT. Meanwhile, those patients also stayed in a good general condition when they experienced the cranial relapse. After reirradiation of WBRT, most patients had better survival outcomes and resolution of neurological symptoms. This conclusion was further supported by more studies. Researchers found that stable extracranial disease was associated with improved survival in patients undergoing reirradiation of WBRT (3, 12, 13). What is more, patients with higher KPS scores had a significantly better prognosis after reirradiation with WBRT (14, 15). In this case, our patient is a male of younger age (<65 years) with a good KPS (KPS >70), control of primary disease, and absence of extracranial metastases. Thus, when he had brain relapse, we chose reirradiation of WBRT for him with a dose of 30 Gy in 15 fractions and a boost for intracranial metastatic sites with 20 Gy in 10 fractions.

Except for the response ratio and survival benefits, the potential toxicity of reirradiation with WBRT is another significant consideration in choosing this treatment option for patients. However, few studies have reported severe toxicities from reirradiation with WBRT till now (4, 5). Radiation-induced brain injury was generally classified into three phases: acute, early-delayed, and late-delayed injury (16), among which we cared about most was the late-delayed injury, commonly observed from 6 months to several years after radiation therapy, because it is usually irreversible and progressive (17). Previous studies reported that cognitive deficiency was one of the most frequent consequences of radiation-induced late-delayed injury (18), occurring in 50%–90% of brain tumor survivors, and it could directly decline the quality of life of the long-term survivors (19). Currently, our knowledge on the mechanisms underlying radiation-induced brain injury is still limited, but it is believed that high radiation doses are responsible for permanent injury, especially the biologically effective dose (BED) (20). What is worth our attention in this case report is that this patient got the cumulative administration dose for the whole brain as high as 70 Gy and a boost for a local site as 30 Gy, without calculating the definite dose of the GK. The BED_cumulative_ of this case is approximately 166.7 Gy (α/β = 3) or 200 Gy (α/β = 2) in total. Even as the total BED is far beyond the conventional limits, this patient ultimately benefited from the repeated radiation of the whole brain and had a good quality of life with just a mild neurocognitive defect. As mentioned above, a break of more than 6 months till the second treatment would be a factor in clinics, as it may allow tissues to recover from occult injury. In this patient, the time interval between two WBRT was as long as 5 years. This could better help to explain his outcomes. Certainly, this time interval was usually accepted in the spinal cord, while rare data were shown in the brain. Considering the similarity in morphological and biological characteristics between the spinal cord and brain, we made this recommendation. What could help us better in the future is that choosing the WBRT technique, no matter for initial or repeated treatment with hippocampal avoidance, could be beneficial to protect neurocognitive function (21).

In fact, during the past decades, some developments have surged into the area of treating multiple BMs. The remarkable expansion of stereotactic radiosurgery (SRS) has been administered to patients with BMs, which was historically been utilized for some benign lesions. SRS is supposed to be particularly suitable for metastatic tumors because most are well-circumscribed. Kann et al. (22) reported that the overall utilization rate for SRS rose from 9.8% to 25.6% from 2004 to 2014. The most widely accepted technique of SRS is GK, while various innovations in linear accelerator (Linac)-based systems are now being well-used worldwide. For a long time, the upper limit of SRS alone without WBRT is generally considered to be four lesions. However, more and more studies have demonstrated that the number could not be the limitation. A trend for patients with ≥5 tumors to be considered for SRS alone is already apparently accepted early in this century (23). The prospective observational JLGK0901 study clearly showed the non-inferiority of SRS with GK for those with 5–10 BMs versus patients with 2–4, no matter in terms of overall survival (OS) or most secondary endpoints (24). Before this, the team of Yamamoto has presented two patients with more than 10 BMs who accepted treatment of GK alone and had a good outcome in the follow-up (25). Meanwhile, due to the technological advancement of Linac, there was a continuous increasing interest in SRS Linac-based applications. In the 1980s, several studies showed similar results in terms of accuracy between 4–10 MV Linac-based SRS and GK (26, 27). Alongi compared the two approaches for BMs in multiple dimensions and found that there were no differences in terms of local progression-free survival, survival rates, toxicity, and cost effectiveness. Even if GK remains superior in terms of deep isotropic dose fall-off out of the target, the integration of IGRT tools for SRS Linac-based treatment guaranteed the option of a frameless SRS safe and reliable similar to GK. Magnetic-resonance-guided radiotherapy (MRgRT) marks the beginning of a new era further (28). Due to the lack of device in this case, we chose WBRT to reirradiate, while a number of retrospective studies have documented re-SRS to be safe and effective (29, 30). Especially with the Linac-based SRS, whether with the non-coplanar mono-isocenter (HyperArc™) technique or the multiple non-coplanar arcs technique, they were demonstrated to improve survival outcomes (31, 32).

In summary, the case report showed that reirradiation of WBRT for patients with multiple BMs even as high cumulative dose still can contribute to good survival and showed slight neurocognitive defects. Therefore, careful selection of the appropriate patient for reirradiation will be needed, and new strategies for BMs would be a better indication. All of these could help to make a proper therapeutic choice.

## Conclusion

Management of BMs has changed substantially in the past 5–10 years, with varied treatments that have a greater focus on disease control and mitigating the effects of treatment, especially on the role of reirradiation of BMs. However, in reality, lots of departments could not afford the new and expensive equipment so that WBRT is still the cornerstone of modern brain metastases treatment. We report the administration of >70 Gy cumulative activity of WBRT without serious toxicity. This case underscores the therapeutic potential of WBRT therapy in metastatic disease and interindividual dose-tolerance variability. This, to our knowledge, represents a high reported value of reirradiation with whole brain radiotherapy for recurrent brain metastases. In an individual attempt to palliate metastatic disease, the high cumulative activity of radiotherapy should not preclude the patient from repeat treatment.

## Data Availability Statement

The raw data supporting the conclusions of this article will be made available by the authors, without undue reservation.

## Ethics Statement

Written informed consent was obtained from the individual(s) for the publication of any potentially identifiable images or data included in this article.

## Author Contributions

ML and TZ conceived and wrote the article. LL and HD managed this patient in clinics. YS did the radiation plan of this patient. JQ collected information of follow-up about this patient. All authors contributed to the article and approved the submitted version.

## Funding

This study was supported by The Project on Science and Technology Situation in Yuzhong District, Chongqing (No. 20190101) without any involvement in writing the manuscript.

## Conflict of Interest

The authors declare that the research was conducted in the absence of any commercial or financial relationships that could be construed as a potential conflict of interest.

## Publisher’s Note

All claims expressed in this article are solely those of the authors and do not necessarily represent those of their affiliated organizations, or those of the publisher, the editors and the reviewers. Any product that may be evaluated in this article, or claim that may be made by its manufacturer, is not guaranteed or endorsed by the publisher.
