# Supplementary material for: Reirradiation of Whole Brain for Recurrent Brain Metastases: A Case Report of Lung Cancer With 12-Year Survival
Source: Front Oncol. 2021 Nov 26;11:780581. doi: 10.3389/fonc.2021.780581 (PMC8660684; doi:10.3389/fonc.2021.780581)
Supplement: Supplementary file 1 [file Table_1.doc]

**神经认知功能测试**

**The report of neuropsychological testing**

**测验结果 Testing scores**：

| 认知 | MMSE： 24 /30  ADAS-Cog ： 6.7 /70 | MoCA： 19 /30 |
| --- | --- | --- |
| 注意和执行力 | TMT  时 间 正确条数 错误  A： 122s 24 00  B： 300s 14 10 | FCSRT顺向： 10 逆向： 04 |
| 语言功能 | BNT：自发命名：正确数 13 错误数： 17  提示命名：正确数 04 错误数： 13 | |
| 学习和记忆能力 | AVLT：正确数 T1： 6 T2： 8 T3： 11  T4(延迟)： 5 T5（线索）： 6 T6（再认）： 14 | |
| 视空间 | CDT： 7 /15 | |
| 精神行为 | GDS： 12 /30 | NPI：严重性： 0 /144  苦恼程度： 0 /60 |
| 日常生活活动能力 | BADL： 6 /30 | IADL： 8 /31 |
| 缺血量表 | HIS： 2 /18 | |
| 临床痴呆评定量表 | CDR： 0.5 /3 | |

MMSE=简易精神状态量表(Mini-Mental State Examination)，ADAS-Cog=阿尔茨海默病评价量表-认知分表(Alzheimer`s disease assessment scal-Cognitive section)，FCSRT=自由、线索和选择性提醒测试(the Free and Cued Selective Reminding Test)，TMT=连线试验(Trail Making Test)，BNT=波士顿命名测试(Boston Naming Test)，AVLT=听觉词汇学习测试(Auditory Verb Learning Test)，CDT=画钟实验(Clock Drawing Test)，GDS=老年抑郁量表(Geriatric Depression Scale)，NPI=神经精神症状问卷(Neuropsychiatric Inventory)，BADL=基本日常生活活动能力(Basic Activities of Daily Living)、IADL=工具性日常生活活动能力(Instrumental Activities of Daily Living)，HIS=缺血量表(Hachinski Ischemic Scale)，CDR=临床痴呆评定量表(Clinical Dementia Rating)

**总体印象**：轻度认知障碍，请结合临床。

Conclusion: mild neurocognitive defects.
